# Adult-Onset Diabetes and Liver Fibrosis as Diagnostic Clues to Alström Syndrome: A Case Report

**DOI:** 10.7759/cureus.102959

**Published:** 2026-02-04

**Authors:** Favour Balogun, Shreya Honarius, Megan Li Yuen Yeoh, Mahmoud Abouibrahim, Mostafa Sayed, Joanne Randall

**Affiliations:** 1 Internal Medicine, James Paget University Hospitals NHS Foundation Trust, Great Yarmouth, GBR; 2 Endocrinology and Diabetes, London North West University Healthcare NHS Trust, London, GBR; 3 Endocrinology and Diabetes, James Paget University Hospitals NHS Foundation Trust, Great Yarmouth, GBR; 4 Gastroenterology and Hepatology, James Paget University Hospitals NHS Foundation Trust, Great Yarmouth, GBR

**Keywords:** alström syndrome, diabetes mellitus, fibrosis, genetic syndrome, liver cirrhosis, metabolic syndrome, multisystem disorder, primary ciliopathy, rare liver disease, resistance to insulin

## Abstract

Alström syndrome (ALMS) is a rare, autosomal recessive condition characterized by progressive multiorgan dysfunction, including vision and hearing loss, obesity, type 2 diabetes mellitus (T2DM), and hepatic and renal impairment. The significant clinical variability and complexity of ALMS often lead to diagnostic delays, with symptoms frequently progressing over many years. This case details a 45-year-old man with a history of early-onset visual impairment and hearing loss (diagnosed at the age of 9-10 years) who subsequently had a late diagnosis of ALMS following the discovery of significant hepatic fibrosis of unknown cause and recent diagnosis of diabetes mellitus without diabetes related antibodies. Given the constellation of symptoms, genetic testing was requested, which ultimately confirmed the diagnosis of ALMS, highlighting how atypical features and delayed recognition can underscore a rare condition with advanced, yet previously unappreciated, organ pathology.

## Introduction

Alström syndrome (ALMS) is a rare autosomal recessive genetic disorder with multisystem involvement, including progressive cone-rod dystrophy and sensorineural hearing loss, early-onset obesity in the first few years of life, insulin resistance, type 2 diabetes mellitus (T2DM), dilated cardiomyopathy, and advancing hepatic and renal dysfunctions. The main pathophysiology includes impaired ciliary function secondary to abnormal ALMS1 protein. Identifying the culprit gene is the main confirmatory diagnostic criterion. To date, 278 genetic variants have been identified as causative for this condition [1,2].

With an estimated prevalence of < 1 in 1,000,000 and fewer than 1200 cases worldwide reported in the literature, ALMS presents unique challenges in diagnosis and management [3]. This report, presenting the case of a 45-year-old male patient diagnosed with ALMS, reinforces the significance of early detection and comprehensive multidisciplinary care in assessing a complex condition, and adds to the literature regarding this under-reported syndrome.

## Case presentation

 A 45-year-old man was referred to the diabetes clinic for diabetes management. After being found with very high HbA1c (152 mmol/L) in 2023, he was started on twice-daily NovoMix-30 insulin. The patient was treated initially as having probable late-onset type 1 diabetes mellitus rather than T2DM, given the marked hyperglycemia, early insulin dependence since diagnosis at age 42, and lacking the typical metabolic features and body mass index (BMI) associated with T2DM. On treatment, his glycaemic control was 79% time in normal range (3.9-10 mmol/L) as measured by a continuous glucose monitoring (CGM) sensor, and his most recent HbA1c was 52 mmol/mol (6.9%), indicating excellent control [4].

Investigating further into his medical history, it was noted that he had significant visual impairment and hearing loss since the age of 9-10 years, and was registered as sight and hearing impaired. Physical examination revealed multiple skin tags in the armpits and around the neck, but no evidence of acanthosis nigricans. As his insulin requirements were relatively low for his age and BMI (28.1 kg/m², overweight), in addition to his sight and hearing impairments, which represent atypical features of diabetes, suspicions were raised for an underlying genetic cause, including the possibility of ALMS [5,6].

**Figure 1:**
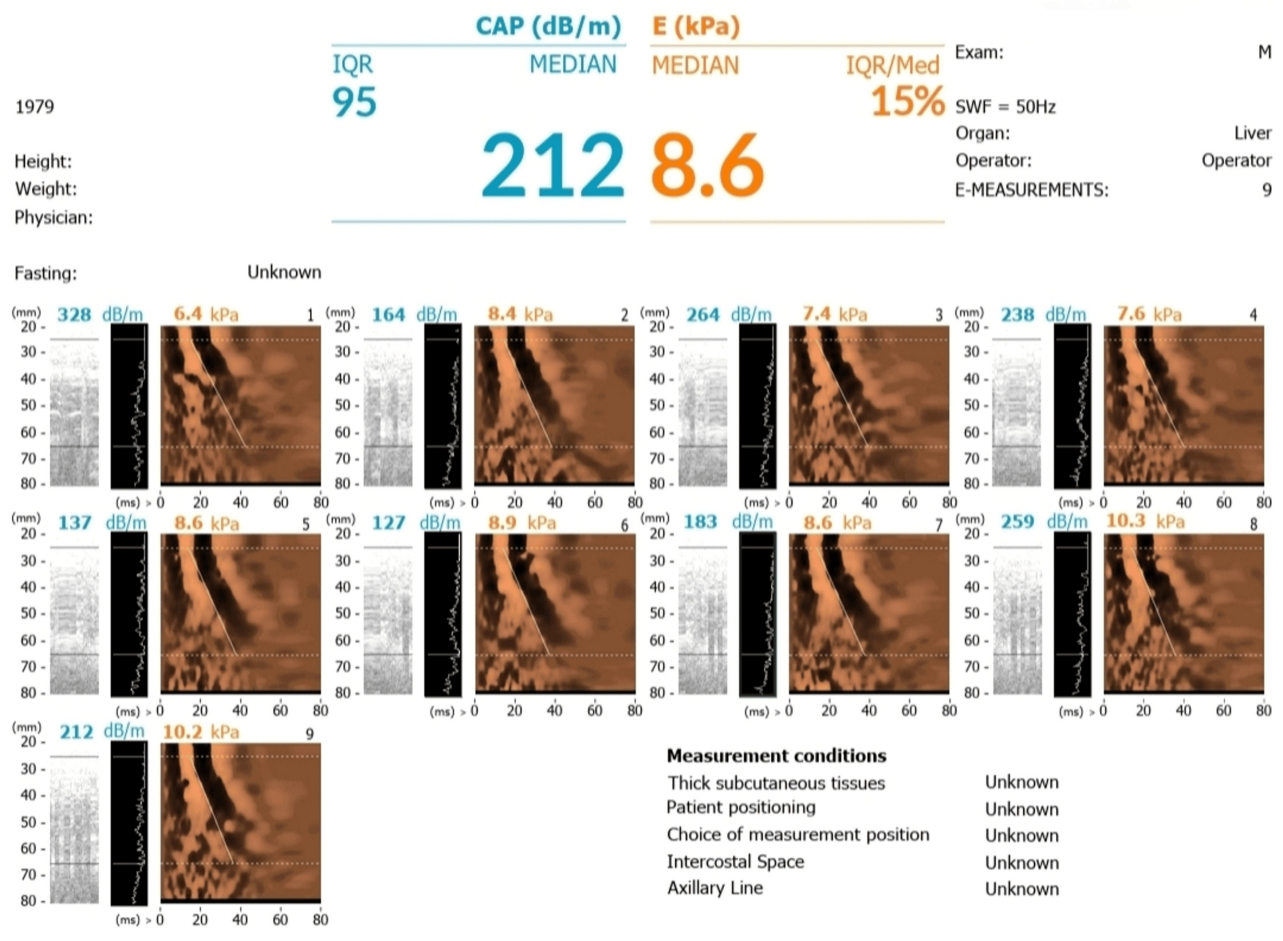
FibroScan demonstrating a median liver stiffness of 8.6 kPa

Following the deranged liver profile with a normal non-invasive liver screen (Table 1), abdominal ultrasound showed mild fatty changes. Subsequently, after hepatology specialist review, FibroScan revealed evidence of fibrosis with a stiffness score of median 8.6 kPa (Figure 1), indicative of moderate fibrosis [7].

**Table 1:** Diabetes-related anti-bodies, liver and renal functions and non-invasive liver screen *Include: anti-mitochondrial antibody (M2), anti-liver kidney microsomal antibody, anti-liver cytosolic antibody, anti SLA/LP, anti M2-3E (BPO), anti SP100 (ANA), anti GP210 (ANA), anti PML (ANA), anti Ro-52. GAD: Glutamic Acid Decarboxylase, IA-2: Insulinoma Associated-2, ZnT8: Zinc transporter 8, HbA1c: Haemoglobin A1c, uACR: urine Albumin to Creatinine ratio, eGFR: estimated Glomerular Filtration Rate, ALT: Alanine Aminotransferase, AST: Aspartate Aminotransferase, GGT: Gamma-Glutamyl Transferase, PLT: Platelets, anti-HAV IgG: anti-Hepatitis A virus Immunoglobulin G, HBsAg: Hepatitis B surface antigen, Anti-HCV: Anti-Hepatitis C virus

Test	Result	Unit of measurement	Reference range
GAD antibodies	Negative	-	-
IA-2 antibodies	Negative	-	-
ZnT8 antibodies	Negative	-	-
HbA1c	52	mmol/mol	-
ALT	150	U/L	0 - 55
AST	39	U/L	11 - 34
GGT	328	U/L	1 - 54
Creatinine	156	umol/L	59 - 104
eGFR	46	mL/min/1.73m^2^	> 90
uACR	3.9	mg/mmol	< 3
PLT	73	x10^9^/L	150 - 410
Anti-HAV IgG	Not detected	-	-
HBsAg	Not detected	-	-
Anti-HCV	Not detected	-	-
Alpha 1 antitrypsin	1.68	g/L	0.9 – 2
Caeruloplasmin	0.23	g/L	0.2 – 0.6
Ferritin	240	ug/L	23 - 300
Liver antibodies line blot*	Negative	-	-

Given the clinical picture of early-onset visual and hearing impairment, diabetes with atypical presentation, liver fibrosis, and renal impairment, genetic testing was performed at Cambridge Genomics Laboratory, which was positive for *the ALMS1* gene variant (c.1732del p.Arg578GIyfs*17). The identified variant results in a frameshift leading to premature truncation of the ALMS1 protein. Based on ACMG/ACGS criteria, it is classified as pathogenic and consistent with a diagnosis of ALMS [8,9]. Subsequently, a referral has been made to a specialised geneticist service, awaiting further review.

**Figure 2:**
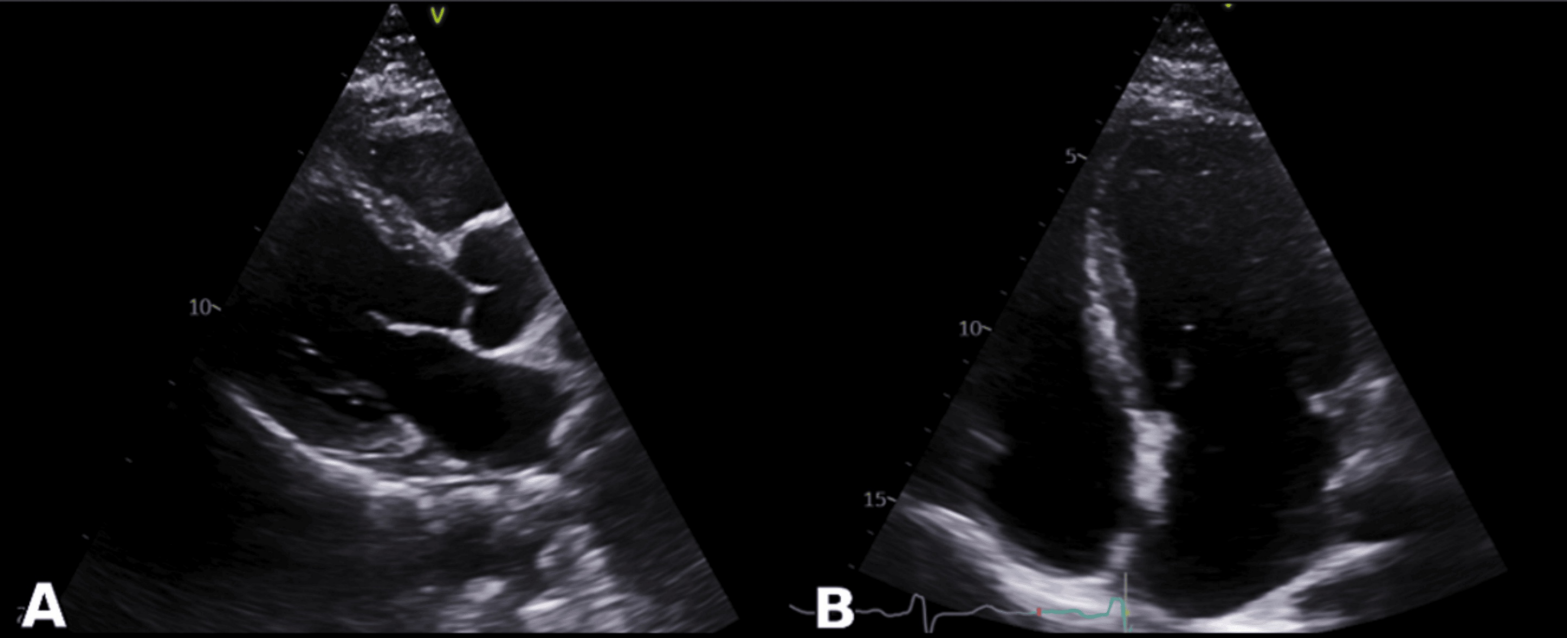
(A) Echocardiographic parasternal long-axis (PLAX) view and (B) Apical four-chamber (A4C) view showing normal left ventricular cavity and normal mitral and aortic valves structure with normal left ventricular wall and septal thickness.

**Figure 3:**
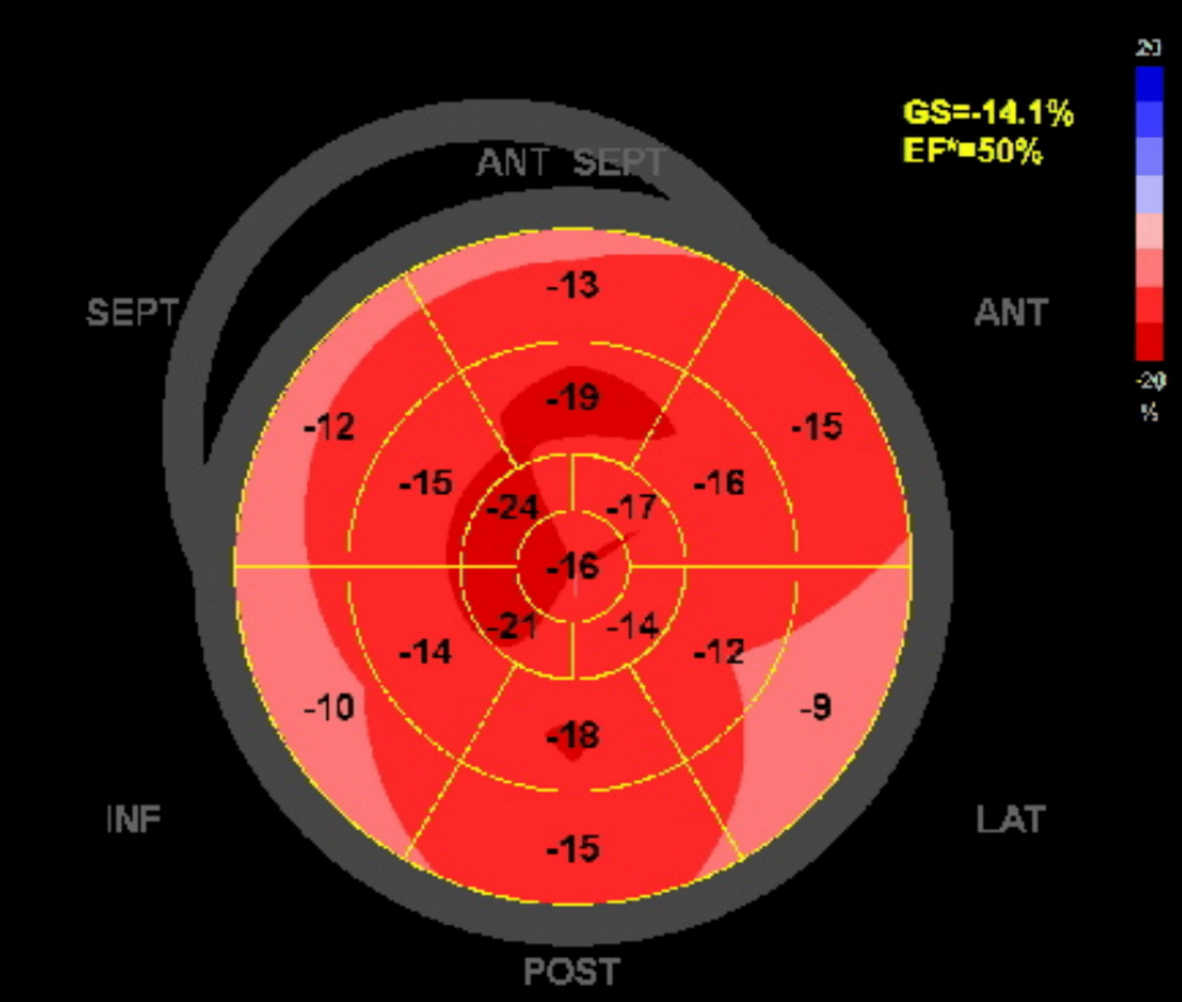
Bull’s eye map shows lateral, inferior and septal mild hypokinesia (light red and pink) with estimated ejection fraction 50%.

Moreover, as part of a comprehensive assessment and screening for multiorgan involvement, transthoracic echocardiography has been arranged, which showed normal valvular structures, chamber sizes, and wall thickness with mildly reduced ejection fraction (EF) at 50% (Figures 2, 3).

## Discussion

Alström syndrome represents a rare but clinically significant monogenic ciliopathy that exemplifies the complex interplay between genetic defects and multisystem organ dysfunction [10].

Genetic basis and pathophysiology

ALMS is caused by autosomal recessive mutations in the *ALMS1* gene, located on chromosome 2p13 [3]. The *ALMS1* gene comprises 23 exons, with pathogenic mutations predominantly found in exons 8, 10, and 16. Mutations of *ALMS1* affect the encoding of a large protein made of 4169 amino acids involved in intracellular transport, ciliary formation and function, and cell cycle control [11]. The syndrome is characterized by ciliopathy, causing diverse clinical manifestations as a result of defective cilia contributing to systemic complications across multiple organ systems [12].

Clinical manifestations

ALMS is characterized by progressive multisystem involvement with significant phenotypic variability even within families carrying identical genetic mutations. Typically, the syndrome manifests in early childhood with cone-rod dystrophy leading to progressive visual loss and eventually blindness. Sensorineural hearing loss progressively develops after the retinal disease, and in one study of 38 ALMS patients, all of the cases had hearing loss preceded by the retinal dystrophy [13,14].

The endocrine manifestations are particularly relevant to our case presentation. Patients develop severe insulin resistance with hyperinsulinemia, typically progressing to type 2 diabetes mellitus. Unlike typical type 1 diabetes, ALMS patients demonstrate negative GAD, IA-2, and ZnT8 antibodies, as observed in this case. The metabolic dysfunction in ALMS is primarily driven by adipose tissue malfunction, which serves as the main driver for insulin resistance and metabolic dysregulation [15,16].

Hepatic, renal, and cardiac complications

This case demonstrates hepatic disease with elevated liver enzymes and evidence of significant liver fibrosis, which represents a well-recognized component of ALMS. The syndrome is associated with a high incidence of non-alcoholic fatty liver disease (NAFLD) that can progress rapidly to fibrosis and cirrhosis [17,18].

The pathogenesis of hepatic fibrosis in ALMS involves variable mechanisms beyond simple steatosis. ALMS1 mutated cells demonstrate overexpression of extracellular matrix components, which leads to cell cycle delay, and show resistance to apoptosis. These cellular characteristics contribute to the extensive fibrosis observed across multiple tissues in ALMS patients [19]. Liver fibrosis in ALMS patients can be assessed non-invasively by FibroScan, which is an ultrasound-based technique that quantifies liver stiffness by measuring elastic shear wave velocity propagation, expressed in kilopascals (kPa). Liver stiffness correlates with histological fibrosis staging (METAVIR (Meta-analysis of Histological Data in Viral Hepatitis) F0-F4), enabling rapid, bedside assessment without biopsy. Fibroscan demonstrates high diagnostic accuracy, with an area under the receiver operating characteristic curve (AUROC) of 0.84 for detecting significant fibrosis [20].

Renal involvement, as evidenced by the elevated creatinine and reduced eGFR in this case, represents another cardinal feature of ALMS. The syndrome is classified as a renal ciliopathy, with ALMS1 protein required for normal kidney ciliogenesis and cellular function. Renal failure is a common cause of mortality in ALMS patients, emphasizing the importance of regular monitoring and appropriate management [21].

Around two-thirds of cases develop dilated cardiomyopathy, fibrosis, and heart failure, which is the most common cause of death; the mechanism of the cardiac disease is not fully understood [22].

Diagnostic challenges

The diagnosis of ALMS presents significant clinical challenges due to its rarity, phenotypic variability, and overlap with other ciliopathies. The condition is frequently misdiagnosed as Bardet-Biedl syndrome (BBS) due to shared clinical features including obesity, diabetes, retinal dystrophy, and hearing loss. However, several distinguishing features can aid in differential diagnosis: ALMS patients typically lack polydactyly, have an earlier onset of symptoms, and demonstrate more severe insulin resistance compared to BBS patients [12].

The absence of pancreatic autoantibodies in the setting of apparent early-onset diabetes should prompt consideration of monogenic causes, particularly when accompanied by syndromic features. The combination of visual and hearing impairment from childhood, diabetes with negative autoantibodies, and evidence of multisystem fibrosis creates a clinical constellation highly suggestive of ALMS [23].

Management and prognosis

Currently, no specific treatments exist for ALMS that can cure the disease, prevent complications, or reverse established organ damage. Management remains primarily supportive, focusing on symptom control and monitoring for complications across affected organ systems. The comprehensive management approach requires multidisciplinary care [22,23].

Diabetes management in ALMS patients requires careful attention to the severe insulin resistance characteristic of the condition in some cases. Standard diabetes medications may be insufficient, and patients often require high-dose insulin therapy or insulin sensitizers. Regular monitoring for diabetic complications, including retinopathy (beyond the primary cone-rod dystrophy), nephropathy, and cardiovascular disease, is essential [23].

Current treatment options for liver fibrosis in ALMS are limited. While liver transplantation has been successful in some cases [24]. Emerging therapies such as antifibrotic agents and targeted gene therapies show promise [25,26]. For instance, PBI-4050, a novel anti-inflammatory and anti-fibrotic agent, is being investigated for its potential in treating the pathological features of ALMS [27].

The prognosis for ALMS remains guarded, with life expectancy rarely exceeding 50 years. Progressive organ failure, particularly involving the liver, kidneys, and heart, contributes to premature mortality. Early diagnosis and appropriate multidisciplinary management may improve quality of life and potentially extend survival, though definitive evidence for improved outcomes with early intervention remains limited [22].

## Conclusions

This case of Alström syndrome in a 45-year-old man highlights the complex, multisystem nature of the disorder and the importance of early detection and comprehensive care. The atypical presentation of new-onset diabetes with significant liver fibrosis and a history of early-onset sensory impairment underscores the heterogeneity of ALMS and the need for considering genetic causes such as ALMS in our differential diagnosis.

Early detection of this condition is important, as it allows for timely intervention and management of various complications. A multidisciplinary approach involving endocrinologists, hepatologists, cardiologists, ophthalmologists, and geneticists is essential for optimal care. While current treatment options for ALMS-related liver disease remain limited beyond liver transplantation, ongoing research into antifibrotic agents such as PBI-4050 and targeted genetic approaches offers hope for future management strategies.
